# Factors Affecting the Quality of Sleep in Children

**DOI:** 10.3390/children8020122

**Published:** 2021-02-09

**Authors:** Ahmad Fadzil

**Affiliations:** Department of Pediatric, Kulliyyah of Medicine, International Islamic University Malaysia, Kuantan 25200, Pahang, Malaysia; drahmadfadzil@iium.edu.my

**Keywords:** children, sleep insufficiency, sleep quantity, sleep quality, affect, factors

## Abstract

Sleep quality is one of the domains of sleep. Having adequate quality sleep is defined as one’s “feeling fresh” after waking-up. Inadequate sleep quality results in sleep insufficiency producing a variety of symptoms and signs. The central nervous system is affected the most in children, although other system too may be involved. Several factors affect sleep quality in children including genetics, sleep habits, medical problems, parents/caregiver factors, screen time and the child’s environment. These factors are inter-related and dynamic. The outcome of sleep insufficiency is many involving neurocognitive and neurobehavior, mood and emotional issues and specific conditions, like pulmonary hypertension, cor pulmonale and obesity. Management should start with proper history taking to identify the multifaceted nature of the condition. Treatment is planned cognizant of the age of the patient and the associated etiological factors, and should involve both the children and their parents.

## 1. Introduction

Sleep is defined as a reversible engagement with unresponsiveness to the external environment, regularly alternating in a circadian manner with engagement and responsiveness [1]. It is one of the human behaviors and is a vital biological need. It is a function of the brain and sufficient sleep is necessary not only to function appropriately when awake but is also vital to stay alive. In humans death is immediate without oxygen, occurs after about 72 h if deprived of water and takes approximately 3–5 weeks without food. Death to occur due to lack of sleep can vary from days to weeks.

## 2. Function of Sleep

There are many theories on the function of sleep based on research in human and animals. These include restoration, evolutionary and adaptive, energy conservation, learning and unlearning [1]. However, the real reason we sleep remains a mystery as do the mystery of our dreams during sleeping, but we do have some understanding about the adverse effects of insufficient sleep to humans including children. Impaired function of several systems especially the central nervous system is noted as a consequence of insufficient sleep in children. This is not surprising as sleep is a function of the brain. The effect on the central nervous system includes the influence to neurobehavior, emotion and mood and neurocognitive functions. The other system that can be adversely affected is the cardiovascular in which sleep insufficiency can result in pulmonary hypertension or systemic hypertension and cardiac impairment including cor pulmonale [2,3,4,5,6,7]. Children with severe sleep insufficiency can also fail to thrive and have delayed developmental milestones [7,8]. Studies have also shown the association of sleep insufficiency with development of type 2 diabetes [9,10]. Sleep insufficiency along with diet and environment also contributed to the current global epidemic of obesity [11].

The signs and symptoms of sleep insufficiency includes persistent sleepiness or hypersomnolence like sleeping at inappropriate time and place, lethargy and the feeling of persistent tiredness, inability to focus and reduced concentration span, deterioration of school performance, poor socialization, hyperactive behavior and low self-esteem. Other possible symptoms are slow motor response to stimuli, irritable and fussiness, snoring, night waking, insomnia, hypertension, orthopnea, decrease effort tolerance, dyspnea, poor or excessive weight gain, abnormal movement during sleep and walking or talking during sleep.

The real prevalence of sleep insufficiency geographically worldwide in children across all ages is unknown. This may be due to difference in needs according to age, socioeconomic and sociocultural status. Even the prevalence between during school holiday and school term can be different. Furthermore, there is no clear definition of sleep insufficiency in children. Nonetheless, sleep insufficiency is not uncommon in the current environment where children sleep duration is shorter due to the demand of modern needs and lifestyle [12].

## 3. Sleep Insufficiency

Sleep insufficiency in children can develop either acutely or manifest chronically. Sleep insufficiency is caused by inadequate sleep quantity (duration), poor sleep quality or both. Sleep quantity and sleep quality are the main domains of sleep and both have to be adequate in order to prevent sleep insufficiency. They are dynamically interrelated and may actually influence each other. The same factors can affect both, even at the same time. However, sleep quality may matter more than sleep quantity [13]. Sleep quantity (duration) is a function of age. Children of different ages need different sleep duration [14]. Conversely sleep quality is defined as adequate when the person feels fresh when waking up from sleep [15]. This definition is somewhat subjective as inadequate sleep due to inadequate sleep duration conceivably will also cause a sense of feeling unfresh when waking up from sleep. In order to be more objective, it is more appropriate to define adequate sleep quality as the ability to maintain age related normal sleep architecture during sleeping. However, because of paucity in the availability and also the complexity to administer polysomnography, it is not feasible to conduct regular polysomnography. Hence a validated definition is unpractical. Furthermore, the relationship between sleep quality and biophysiological changes recorded by polysomnography is still not fully clarified [16]. Specific biophysiological changes recorded such as significant arousals, hypercapnia, hypoxia, respiratory event, sleep efficiency and fragmented sleep can indicate poor sleep quality. There is a need to have an objective definition of sleep quality, so that more studies that focus on sleep quality rather than sleep quantity could be conducted, unlike the current trend, which focuses more on sleep quantity. There is limited research that particularly focuses on sleep quality in children because of children’s limited ability to be expressive due to age. How children feel is dependent on parents reporting and interpretation of children behaviors as a surrogate. Hence, it is not easy to exclude the other causes of signs and symptoms of sleep insufficiency in children.

**Figure 1:**
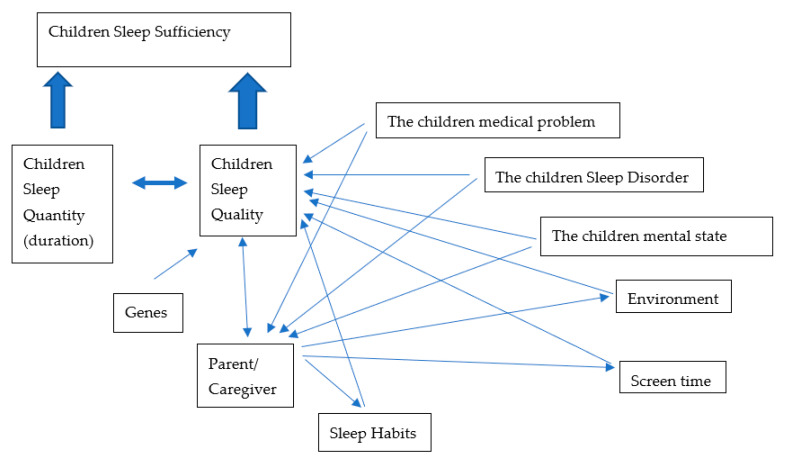
Factors that affect sleep quality and the interactions between factors.

Numerous causes affect sleep quality, either directly or indirectly and usually these multiple factors can interact at any given time contributed to poor sleep quality. These factors could be genetic, parents or caregiver issues, sleep habits, environment influences including excessive screen exposure, sleep disorders and mental and medical problems (Figure 1).

## 4. Factors Affecting Sleep Quality

### 4.1. Gene

How and to what extent the gene regulates one’s sleep is still not fully understood. Studies using questionnaires and actigraphy suggest that genes may have a direct influence in sleep quality by influencing certain sleep traits such as sleep duration, insomnia and chronotype [17,18,19,20]. The individual sleep duration needed may be governed by certain genetic loci [21]. There is a significant interaction between children sleep duration and polymorphism of 5-HTTLPR genotype, and its effect on the negative behavioral score in the first 3 years of life [22]. The disparities in the development of sleep in many adolescents who have shifted sleep phase, lark versus owl (extreme chronotype), variability in the time of melatonin secretion in individual and variation in the circadian cycle showed involvement of genes in sleep. As in many other diseases, the interaction between gene and environment in the phenotype manifestation is not fully understood. However, a study on twins suggested that genes might not be as influential as the environment [23].

Gene is also involved indirectly in diseases that have genetic preponderance such as in atopic disease like asthma, allergic rhinitis or eczema. Children with severe or poor control of these conditions have poor sleep quality due to the persistent symptoms they experience during sleep.

### 4.2. Parent or Caregiver

Parents have a very intimate relationship with their children, and it is a mutual relationship. Parents can affect and influence their children’s sleeping habits and vice versa [24,25]. Parents not only provide physical care but also emotional support to their children. Parent’s actions are crucial in the management of their children’s behavior, time, screen time exposure and also sleep. Their activities contribute to sleep onset and sleep maintenance especially in young children [26]. The younger the children, the more dependent they are on their parents. It is the parents’ responsibility to instill good sleep hygiene as early as possible in their children and to create a conducive environment for sleeping. Children with knowledgeable parents showed a healthier sleeping habit [27]. Parents with better sleep knowledge, higher socioeconomic status and higher education levels are more likely to report earlier bedtimes, earlier wake times and more consistent sleep routine of their children that is suggestive of possessing better sleep quality [28]. The involvement of the parents is crucial in the management of most of their children’s sleep difficulties. Failure of parents to adequately participate in the treatment of their children’s sleep issues will result in the persistence of these issues and can eventually affect their sleep quality. Some studies have shown that parental medical problems, sleep problems and the state of parents’ mental condition can affect their children’s sleep. It has been noted that depression in parents, could result in sleep insufficiency in their children [29,30]. Certain behavior in parents, such as abusive and frequent altercations among parents can create emotional problems in their children leading to impairment of the child’s sleeping quality [31].

### 4.3. Sleep Disorders and Medical Problem

There are many sleep disorders involving children and these can affect their sleep quality [32]. Conversely poor sleep quality may also aggravate and perpetuate sleep disorders in the children. These sleep disorders include sleep-disordered breathing such as obstructive sleep apnea syndrome or sleep hypoventilation syndrome, the parasomnias, circadian rhythm sleep–wake disorder, sleep related rhythmic movement disorder, sleep related enuresis, bedtime resistance and restless leg movement [33]. These disorders can increase sleep latency and reduce sleep duration, result in frequent awakening leading to fragmented sleep, cause fluctuations in sleep stage ratio, reduce sleep efficiency and also trigger alterations in cerebral blood flow during sleep.

Children’s own medical condition can affect their sleep quality. In children with acute medical conditions, the effect on their sleep quality is dependent on the severity of the illness and is usually a temporary event. Most of the children will recover when the acute illness resolves. On the contrary, they may have persistent poor sleep quality in chronic illnesses. This is evident in conditions such as poorly controlled asthma, allergic rhinitis or atopic dermatitis. Children with chronic pain as in juvenile rheumatoid arthritis and children with oncology related illness too might have persistent poor sleep quality. Many of them have difficulty in initiating and maintaining sleep [34,35,36,37]. Children with mental disturbances like anxiety, stress and worry too experience problems during sleep [38]. The stressors for these emotional disturbances are diverse and include school demands, social life interactions and family dynamics.

Children with autistic spectrum disorders and attention deficit hyperactive disorders experience poor sleep quality and this is an integral part of the diseases. Hence treating the disorders will alleviate their sleep problems [39,40]. Thirteen percent of the cerebral palsy children have total abnormal scores and another 40% have at least one abnormal score in one of the domains of sleep disturbance scale for children. A significant number of children in this group have sleep problems as evident from these findings and it is most likely their sleep quality [41].

A recent longitudinal study demonstrated sleep quality to be affected in children during the current COVID-19 epidemic. It was significantly affected at the initial phase of the lockdown due to the abrupt change in behavior and social norm [42].

### 4.4. Sleep Habit, Sleep Environment and Medications

Good sleep habits should be instilled from infancy and should be an essential part of a child’s development. A significant number of infants with sleep problems continue to have sleep problems when they reach preschool age [43]. Sivertsen et el. showed an association between sleep problems in toddlers when at the age 18 months with behavioral and emotional disturbance when they reach 5 years of age [44]. Following good sleep habits and practices can alleviate sleep problems in children [45,46]. Good sleep habits include consistent sleep routine, avoidance of active and stimulating activities before sleep, utilizing only the bed for sleeping, adhering to consistent sleep and wake up time, and acceptable difference between weekday and weekend sleep patterns. These will promote quality sleeping. Sleep habits should be age appropriate. Children should sleep in a conducive environment suitable for sleeping. This entails appropriate temperature, proper lighting and minimal noise level [47]. The increase sleep-onset latency and a noisy, not well-darkened room are predictive of increased odds of having sleep problems in children [48]. In general, children especially those with sleep problems should avoid any stimulants prior to sleeping. Drink or food containing caffeine should not be consumed for a few hours before sleep [49,50]. Many medications affecting the central nervous system and sleep architecture should be avoided. This also includes substances that cause addiction and abuse [33]. These substances impair sleep quality by disrupting the awake–sleep pattern and brain function [33].

### 4.5. Screen Exposure

Discretionary screen time results from prolonged exposure to multiple devices such as mobile phones, tablets, televisions, computer monitors and game consoles. It is a worldwide phenomenon and screen culture is an integral part of human culture and behavior. It is very difficult to avoid not using devices without a screen in everyday life activities. However, overuse and psychological dependent on these gadgets are signs of addiction. Sleep is affected by the overuse of screen-time, causing sleep restriction. Time allocated for sleep is used for interacting with these screens [51], and this practice affects the sleep wake cycle. Screen light stimulates the brain and suppresses melatonin production resulting in an increased sleep latency [52]. The presence of television in the bedroom is not suitable for a wholesome and adequate sleep [53].

## 5. Management

It is very important for health practitioners to recognize the symptoms and signs of sleep insufficiency. Many parents and their children do not visit doctors with complaints pointing to a particular sleep problem unless they have a specific sleep disorder where the presentation is apparent. The examples are the parasomnias, movement disorders or NREM related sleep disorder. It is crucial to emphasize the importance of an accurate history to identify the symptoms and signs and their relationship to sleep to be recognized. The history includes sleep history, dietary habits, schooling and social history, medical history, exercise routines, family and parental issues, ingestion of medication or any stimulants before sleep, substance abuse and importantly screen time. All this information is crucial to identify any potential sleep problems in the child. Physical examination findings will depend on the causes and in some situations can invariably be normal. These children should have a properly documented sleep diary that needs to be analyzed thoroughly. Specific investigations will depend on the respective system involved such as cardiovascular or endocrine system. Polysomnography is helpful in certain conditions such as sleep-disordered breathing, rapid leg movement or narcolepsy. In many instances, a detailed and accurate history is adequate to identify the factors affecting sleep quality and diagnose the disorder. The treatment of the child concurrently with their parents counseling should be designed befitting the diagnosis and the age of the patient. Practical methods to mitigate identifiable factors such as improving parental education on children’s sleep, introduce good sleep hygiene, manipulating the sleeping environment, controlling and treating of chronic illnesses, proper use of gadgets, behavior modification should be addressed. Specific treatment for various sleep disorders should be instituted if available. Occasionally, medications, surgical interventions and machines may be needed to help the child to have adequate sleep.

## 6. Conclusions

Sleep quality is an important factor causing sleep insufficiency. Multiple factors can affect children sleep quality, and these factors tend to interact within a same time frame. Health practitioners have to recognize these factors and adopt a holistic approach in managing this condition. Although sleep quality may be more important in influencing sleep sufficiency, specific studies in children are limited due to the deficiency of their expressive abilities. Thus, a more objective definition is needed.

## Data Availability

The author is the sole person who conceived, did the research and wrote the article.
